# A Generic Image Processing Pipeline for Enhancing Accuracy and Robustness of Visual Odometry

**DOI:** 10.3390/s22228967

**Published:** 2022-11-19

**Authors:** Mohamed Sabry, Mostafa Osman, Ahmed Hussein, Mohamed W. Mehrez, Soo Jeon, William Melek

**Affiliations:** 1 Autonomous Mobility and Perception Lab (AMPL), Universidad Carlos III de Madrid (UC3M), 28911 Leganes, Spain; 2 Mechanical and Mechatronics Engineering, University of Waterloo, Waterloo, ON N2L 3G1, Canada; 3 Intelligent Driving Function Department, IAV GmbH, 10587 Berlin, Germany

**Keywords:** visual odometry, image processing pipeline, computer vision, robot operating system (ROS)

## Abstract

The accuracy of pose estimation from feature-based Visual Odometry (VO) algorithms is affected by several factors such as lighting conditions and outliers in the matched features. In this paper, a generic image processing pipeline is proposed to enhance the accuracy and robustness of feature-based VO algorithms. The pipeline consists of three stages, each addressing a problem that affects the performance of VO algorithms. The first stage tackles the lighting condition problem, where a filter called Contrast Limited Adaptive Histogram Equalization (CLAHE) is applied to the images to overcome changes in lighting in the environment. The second stage uses the Suppression via Square Covering (SSC) algorithm to ensure the features are distributed properly over the images. The last stage proposes a novel outliers rejection approach called the Angle-based Outlier Rejection (AOR) algorithm to remove the outliers generated in the feature matching process. The proposed pipeline is generic and modular and can be integrated with any type of feature-based VO (monocular, RGB-D, or stereo). The efficiency of the proposed pipeline is validated using sequences from KITTI (for stereo VO) and TUM (for RGB-D VO) datasets, as well as experimental sequences using an omnidirectional mobile robot (for monocular VO). The obtained results showed the performance gained by enhancing the accuracy and robustness of the VO algorithms without compromising on the computational cost using the proposed pipeline. The results are substantially better as opposed to not using the pipeline.

## 1. Introduction

Throughout the past few years, interest in autonomous robotic systems has increased drastically. One of the key modules required to achieve complete autonomy is localization, which is the ability of a mobile platform to determine its position and orientation. Currently, several sensors are used to achieve localization, including LiDAR [1], radar [2], Global Positioning System (GPS) [3], Inertial Measurement Unit (IMU) [4], wheel encoders [5], and cameras [6].

One of the common methods for localization using cameras is through Visual Odometry (VO) [7,8]. VO estimates the ego-motion of a camera by determining the incremental motion between the successive camera frames. Like other odometry methods (wheel encoders, LiDAR, and so on), VO relies on integrating the incremental motion between successive frames to compute the overall trajectory of the camera, leading to drift errors over long distances.

To avoid drift errors, VO is usually integrated into a Simultaneous Localization and Mapping (SLAM) system with a loop-closure module to correct the drift error [9,10,11]. Although using SLAM is beneficial for acquiring a map of the environment, it incurs unnecessary computational costs when the map is already known. A better solution could be using an accurate low-drift odometry module alongside localization in a pre-acquired map [12].

Several VO algorithms were developed in the literature aiming to increase pose estimation accuracy. Those algorithms are classified by the type of camera used in the motion estimation as monocular, stereo, and RGB-D VO. Alternately, they can also be classified by the method of motion estimation into feature-based and direct VO [13].

Monocular VO uses images captured by only one camera to determine its trajectory. This technique usually relies on Structure from Motion (SFM) [14,15,16,17]. With one camera, the motion of the robot can be captured up to an unobservable scale. This scale can then be determined through the use of an external velocity measurement from a wheel encoder or an IMU. Several researchers developed methods for estimating the scale of monocular VO without the use of external sensors, such as [18,19].

Lately, researchers have started developing deep-learning techniques for monocular VO [20,21]. Unlike the monocular VO, the stereo and the RGB-D VO estimate the full pose of the vehicle without any external measurements [22,23,24].

All three categories of VO can be either direct or feature-based approaches. On the one hand, the feature-based method relies on visual features for calculating the transformation between consecutive frames. For the detection of such features, a feature detector and descriptor are used such as Scale Invariant Features Transform (SIFT) [25], Speeded-up Robust Features (SURF) [26], or Oriented FAST and Rotated BRIEF (ORB) [27] (see [28] or [29] for a comparison between the different detectors and descriptors). On the other hand, direct approaches compute the relative transformation between frames based on the whole-image intensities [30,31].

Although such VO techniques are well-designed, VO algorithms have a drawback in that they rely on integration, which may suffer from drift errors due to, for example, false-matched features, bad lighting and illumination problems, random noise, or motion bias [32].

To overcome the above-mentioned issues, this paper proposes a generic modular image processing pipeline to enhance the accuracy and robustness of feature-based VO algorithms, independent of the camera type (monocular, RGB-D or stereo vision). The proposed pipeline includes additional filtration and pre-processing stages through Contrast Limited Adaptive Histogram Equalization (CLAHE) with adaptive thresholding to dynamically overcome variable lighting conditions. Additionally, the Suppression via Square Covering (SSC) is used to make the extracted features more equally distributed across the image to reduce the bias in the motion estimation [33]. Finally, a novel outlier rejection algorithm called the Angle-based Outliers Rejection (AOR) is proposed to reject false-matched features, as well as features captured on a moving object in the scene. The pipeline is integrated into a monocular, RGB-D, and stereo VO and validated using KITTI [34] and TUM datasets [35], as well as experimental sequences generated by an omnidirectional mobile robot.

The contribution of this paper is integrating the CLAHE filter, the SSC algorithm, and the proposed AOR algorithm into the VO. The results show that the three stages play an integral part in enhancing the performance of VO while overcoming the drawbacks of every individual stage.

The remainder of this paper is organized as follows, Section 2 discusses the related work, and Section 3 explains the proposed pipeline with the different filtration steps. The experimental work is presented in Section 4, which also includes the implementation details, along with the used datasets and the evaluation metrics. Section 5 presents the results and discussions. Finally, Section 6 provides concluding remarks and future works.

## 2. Related Works

### 2.1. Image Filtration

Several works have addressed the problem of noise in images for VO enhancement. The noise may be attributed to poor lighting conditions caused by light source flare, random visual sensor noise, or other noise sources [36].

In [37], a direct VO algorithm using binary descriptors was used to overcome poor lighting conditions. The authors showed that the algorithm performed in a robust and efficient way even under low lighting conditions. This was accomplished by the illumination invariance property of the binary descriptor within a direct alignment framework. The VO algorithm proposed therein is a direct method, which is usually more computationally expensive compared to feature-based VO.

In [38], a method for reducing drift in VO is introduced. The authors develop a new descriptor called the SYnthetic BAsis (SYBA) descriptor to reduce false-matched features. This is accomplished with the help of a sliding window approach. The features matching step is applied to features in a window instead of matching features between two consecutive frames. Although a sliding window approach can, in fact, increase the accuracy of the feature matching step, it will also significantly increase the computational cost of the matching task.

In [39], a robust feature-matching scheme was combined with an effective anti-blurring frame. The algorithm uses the singular value decomposition to mitigate the effect of blurring due to vibrations or other factors.

In [40], a stereo visual SLAM algorithm was proposed, which uses CLAHE to locally enhance the image contrast and obtain more feature details. The CLAHE-enhanced SLAM algorithm was compared to the results of a VO enhanced by a conventional histogram equalization and the results of ORB-SLAM2 [11]. The results showed a superior performance of the CLAHE-enhanced algorithm compared to the other algorithms. Furthermore, in [41], a robust VO for underwater environments was proposed. In order to overcome the turbid image quality of underwater imaging, the authors used CLAHE for contrast enhancement. The authors showed that the use of CLAHE resulted in brighter and larger visible regions. As a result, unclear structures were significantly reduced.

Therefore, in this paper, CLAHE is selected as a pre-processing stage for the camera frames to overcome the effect of poor lighting conditions.

### 2.2. Non-Maximal Suppression

Non-maximal suppression can be used to avoid poor distribution of features over the image, which leads to poor VO performance and motion bias. Several non-maximal suppression algorithms were used in VO [42,43]. In [44], a feature descriptor was proposed to facilitate fast feature matching processing while preserving matching reliability. The authors chose to use the FAST (Features from Accelerated Segment Test) detector [45] along with a non-maximal suppression algorithm.

In [46], a stereo/RGB-D VO was proposed for mobile robots. Therein, the authors used the adaptive non-maximal suppression introduced in [47] to enhance the performance of the feature detector algorithm BRIEF (Binary Robust Independent Elementary Features) [48] by ensuring uniform distribution of features over the image.

In [33], three new and efficient adaptive non-maximal suppression approaches were introduced, which included the SSC algorithm. The positive impact of the three algorithms on visual SLAM was demonstrated. Authors in [33] showed that the output of the three algorithms is visually and statistically similar; however, SSC showed lower computational costs, which suggests that it is more suitable for real-time applications such as VO.

Although the authors of [33] showed the effect of the SSC on the enhancement of the output of a visual SLAM algorithm, to the best of the authors’ knowledge, the SSC algorithm was not used in any other VO or visual SLAM algorithm afterward. In this paper, SSC is selected as an additional stage for feature detection and matching to avoid the bias in the motion estimation due to poor distribution of the features.

### 2.3. Outliers Rejection

Feature-based VO relies on feature detection and matching for motion estimation. Commonly, the feature matching algorithms generate a considerable amount of false-matched features [7]. This is mainly due to the limitation of using local feature matching. These false-matched features lead to the increased error in motion estimation or the complete divergence of the VO output, as well as increased computational costs. Several works in the literature addressed this problem. In [49], an iterative outlier rejection scheme for stereo-based VO was proposed. The proposed algorithm was designed to improve the VO motion estimation for high-speed and large-scale depth environments.

In [50], a stereo VO was proposed that relies on using reference frames instead of all frames. This was accomplished by first selecting the stable features from a frame using quad-matching testing and grid-based motion statistics. Afterward, the features in this frame were matched to the features in a reference frame (instead of the previous frame), which contained the stable features found in the current frame.

A commonly used outlier rejection approach is the Random Sampling Consensus (RANSAC). RANSAC is an iterative outlier rejection algorithm, which relies on the computation of model hypotheses, from a randomly selected set of the matched features, followed by the verification of the hypotheses using the rest of the matched features [8]. In [51], a stereo VO algorithm was proposed, which uses a RANSAC-based outliers rejection along with an iterated sigma point Kalman filter to achieve robust frame-to-frame VO performance. Although RANSAC is effective in removing outliers, the iterative process sometimes results in poor performance due to a large number of iterations for convergence. Furthermore, if the number of outliers in the matched points is large, this may lead to wrong convergence entailing incorrect motion estimation.

Hence, in this paper, a non-iterative outliers rejection algorithm is proposed, which relies on the angular distance of the matched features in the matched frames. The algorithm can be incorporated before RANSAC in order to reduce the number of iterations required for convergence and thus increase the overall accuracy of motion estimation while reducing the computational cost of the algorithm.

## 3. Proposed Pipeline

**Figure 1:**
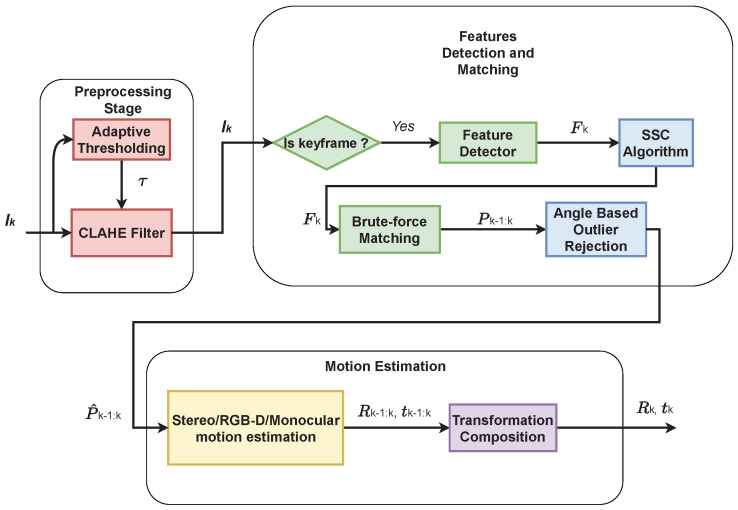
Proposed pipeline for the robust feature-based VO with the added filtration stages highlighted in red.

In this section, the components of the proposed image processing pipeline are introduced. The flow chart of the pipeline is shown in Figure 1.

### 3.1. Image Pre-Processing

**Figure 2:**
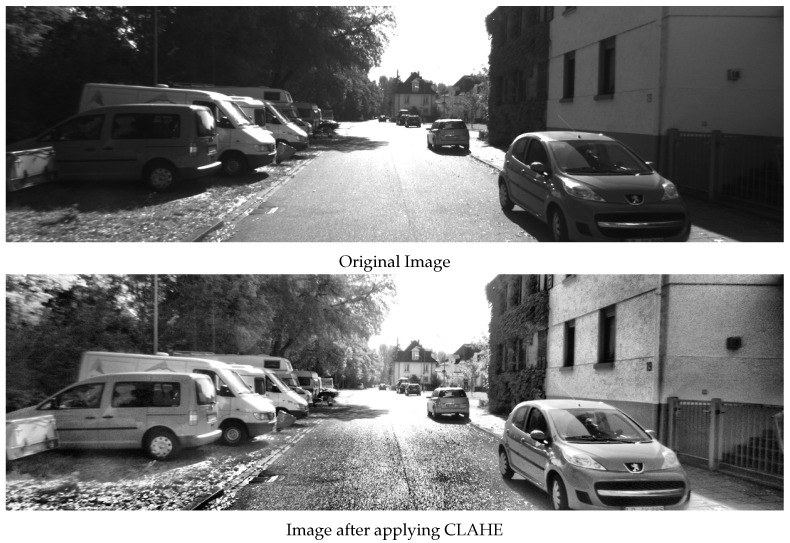
The effect of CLAHE filter on the image. Top: the image before applying CLAHE, Bottom: the image after applying the CLAHE algorithm with the adaptive thresholding. The image was taken from the KITTI dataset.

The pre-processing stage consists of applying a simple blurring filter to remove some noise in the image, followed by applying an Adaptive Histogram Equalization (AHE) technique, namely CLAHE [52]. CLAHE is applied to each input frame to enable the feature detector to find a sufficient number of features per frame. Although traditional AHE techniques tend to over-amplify the noise in the nearly constant regions in an image, the CLAHE filter prevents this over-amplification by limiting the histogram values. The effect of the CLAHE is shown in Figure 2.

Moreover, to ensure that the CLAHE filter adapts to different lighting conditions, the threshold value of the CLAHE is made adaptive to the ratio between the minimum, maximum, and the median of the intensity values in the frame, as presented in (1). The adaptation of the threshold value enables the CLAHE filter to adapt to different lighting conditions during the mobile platform operation and to avoid deterioration of the VO performance caused by too high or too low brightness in the images. Specifically, the contrast at which the CLAHE filter clips the histogram is computed as

(1) $$\tau_{k}=\frac{\max\left(I_{k}\right)-\min\left(I_{k}\right)}{\mathrm{median}(I_{k})}$$

where $\tau_{k}$ and $I_{k}$ are the contrast threshold for the CLAHE filter and the 2D image data at the *k*-th time step, respectively.

An example of the output of the CLAHE filter is shown in Figure 2. The effect of the sun can be seen in the original image, which leads to bright regions in the top middle of the image and dark regions on the left and the right of the image. Applying CLAHE results in decreasing the effect of the sunlight on the image and increasing the amount of extractable information, which can then be used by the feature detector algorithm to capture more stable features from the image.

### 3.2. Features Detection and Matching

After the image pre-processing, the current frame $I_{k}$ is passed to a feature detector. The extracted set of features, denoted by $F_{k}$, is then matched with the set of those from the reference frame $F_{k-1}$. Then, the set of matched features $P_{k-1:k}$ is used for estimating the incremental motion between the two frames $I_{k-1}$ and $I_{k}$.

One of the causes of error in the motion estimation is the nonuniform distribution of features associated with the image [32,46]. Another cause of poor motion estimation is the presence of a high number of outliers in the detected and matched features. Therefore, in this paper, we added the SSC as well as the proposed AOR steps to the proposed pipeline.

#### 3.2.1. Suppression via Square Covering

Before passing the feature set $F_{k}$ to the feature matching algorithm, the features are first passed to the SSC [33] algorithm to make sure the captured features are homogeneously distributed over the whole captured image $I_{k}$.

The SSC algorithm is an approximation of the Suppression via Disk Covering (SDC). It relies on an approximate nearest neighbor algorithm, which uses a randomized search tree. In contrast, the SSC achieves comparable results with a single query operation per search range guess. Accordingly, the SSC has better efficiency and scalability than the SDC. In addition, SSC applies a square approximation for the SDC to avoid computing the Euclidean distance between a large number of features. This allows the SSC algorithm to execute in a runtime with lower complexity as the number of features increases.

**Figure 3:**
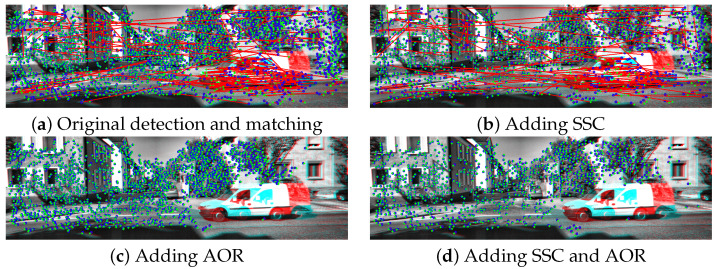
The effect of SSC and AOR on the features detection and matching. The previous frame is shown as the red component of $I_{k-1}$, and the current frame is shown as the blue component of $I_{k}$. The green crosses (+) and the blue circles (∘) represent the features $F_{k-1}$ and $F_{k}$, respectively. Finally, the red lines represent the matched pairs in $P_{k-1:k}$. Each of the images represents the following: (**a**) the original feature pairs through using the SURF detector and a brute-force matching algorithm, (**b**) shows the feature pairs after adding the SSC algorithm only. (**c**) shows the feature pairs after adding the AOR algorithm only, and finally (**d**) shows the feature pairs after adding both SSC and AOR. The majority of the outliers were removed due to the large difference between the angles, as illustrated in Figure 4.

The effect of using the SSC algorithm is shown in Figure 3. Figure 3a shows the original output of the SURF feature detector, where the feature density is higher in the top right region of the image. As shown in Figure 3b, after applying the SSC, the feature distribution across the image is almost the same (except for regions that did not contain any features).

Although several feature matching algorithms were introduced in the literature [53], those algorithms generate a considerable amount of false-matched points (as can be seen in Figure 3a,b. Motivated by this issue, in this paper, a novel outlier rejection algorithm is introduced and integrated into the overall VO pipeline to remove those false-matched points.

#### 3.2.2. Angle-Based Outliers Rejection (AOR) for Feature Matching

To filter the produced matched points from outliers, a new outlier rejection algorithm called the AOR is proposed. In addition to removing false-matched features, the AOR can remove features that do not belong to the ego vehicle motion in the scene. This is applied before the motion estimation stage (RANSAC/LMEDS) in order to reduce the number of iterations required for convergence and thus increase the overall accuracy of motion estimation while reducing the computational cost of the algorithm.

**Figure 4:**
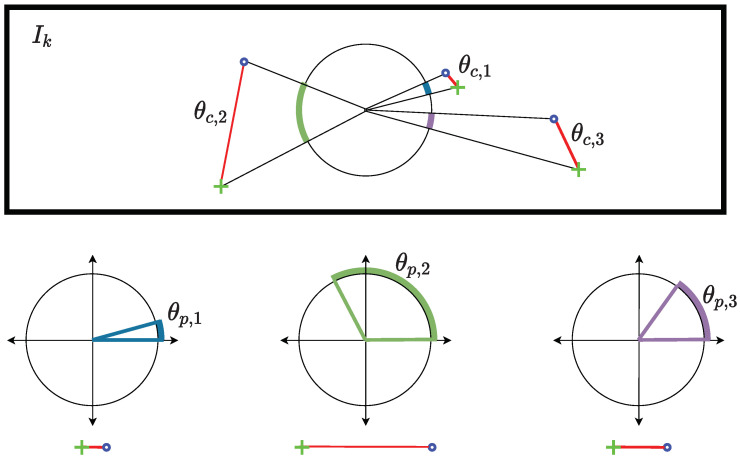
Visual illustration of $\theta_{c}$ and $\theta_{p}$ for three different feature pairs in an image.

Usually, during the motion of the camera, the further the feature from the vanishing point of the image, the more the feature moves. Although the amount of movement of a feature in the successive images is different depending on its position, it should be comparable to the motion of other features. False-matched points tend to show a larger amount of feature motion through the image, as shown in Figure 3a. The AOR uses the distance traveled by the feature in the successive images along with the actual position of the feature in those images to remove false-matched points, as illustrated in Figure 4.

Figure 4 illustrates the two metrics used by the proposed AOR. AOR can be divided into two steps. First, the angle $\theta_{c}$ between the lines drawn from the center of the image to the feature (shown in Figure 4) in $I_{k-1}$ and $I_{k}$ is simply calculated as [54].

(2) $$\theta_{c,i}=\arccos\frac{x_{k-1,i}\cdot x_{k,i}+y_{k-1,i}\cdot y_{k,i}}{\sqrt{x_{k-1,i}^{2}+y_{k-1,i}^{2}}\cdot \sqrt{x_{k,i}^{2}+y_{k,i}^{2}}},$$

where $\left(x_{k-1,i},y_{k-1,i}\right)$ and $\left(x_{k,i},y_{k,i}\right)$ are the coordinates of the *i*-th feature in the frames $I_{k-1}$ and $I_{k}$, with respect to the center of the image, respectively. Notice that $\theta_{c}$ represents the amount of feature motion, irrespective of its position in the frame. $\theta_{c}$ for different feature positions is illustrated in the top plot of Figure 4.

Second, the Euclidean distance traveled by the feature projected on a reference circle, as shown in the bottom plot of Figure 4, and the corresponding angle $\theta_{p}$ is calculated as [55]:

(3) $$E_{i}:={∥\left[\begin{array}{c} x_{k,i}\\ y_{k,i} \end{array}\right]-\left[\begin{array}{c} x_{k-1,i}\\ y_{k-1,i} \end{array}\right]∥}_{2},$$

(4) $$\theta_{p,i}=\frac{E_{i}}{R}$$

where *R* is the radius of the reference circle. Notice that *R* determines the sensitivity of the values of $\theta_{p}$. The larger the radius, the smaller the angle for larger Euclidean distances. The radius can be calculated as,

(5) $$R=\sqrt{\frac{c_{x}^{2}+c_{y}^{2}}{\zeta }},$$

where $c_{x}$ and $c_{y}$ are the centers of the image, and ζ is a parameter that controls the size of the radius. During the experimentation, the best results were obtained by ζ=8; however, different values may work better for different conditions. Notice that here we assume that the vanishing point is in the center of the image. Although this is not generally true, in the case of a ground vehicle motion, such an assumption did not affect the results acquired by the algorithm. Furthermore, through testing the algorithm with a vanishing point extraction algorithm [56], the output was similar in accuracy. Therefore, we chose to omit such a step to achieve faster VO pipeline.

Using the two angles $\theta_{c}$ and $\theta_{p}$, a score *S* is computed for each feature as:

(6) $$S_{i}=\left|\theta_{c,i}\theta_{p,i}\left(\theta_{c,i}-\theta_{p,i}\right)\right|.$$

Notice that for every matched feature, the greater $\theta_{p}$ and $\theta_{c}$ values and the difference between them, the larger the score AOR yields.

Finally, a feature is selected as an inlier, if its AOR score value is less than a threshold η calculated as

(7) η=c·median(S),

where S is the score set of the matched features, and c>1 is a parameter, which is set in this paper to 2 (through tuning). The overall AOR algorithm is summarized in Algorithm 1.

Figure 3c shows the effect of AOR on the detected features. By using AOR, all the outliers, which are present in Figure 3a, are removed, and only the true features describing the motion of the camera remain. Furthermore, notice the effect of the AOR algorithm in removing the features detected on the moving vehicle present in the image since the motion of such features does not agree with the motion of the remaining features. Figure 3d shows the effect of both SSC and AOR on the image, where the inliers remaining in the image are better distributed due to the SSC effect.
**Algorithm 1:** AOR Algorithm**Set** R and η**for**$p_{i}\in P_{k-1:k}$**do**
   
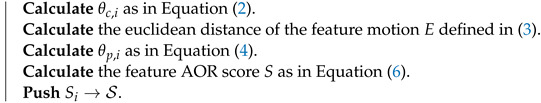

**end**(**Calculate**η as in Equation (7).**for**$p_{i}\in P_{k-1:k}$**do**
   
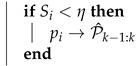

**end**(where ${\hat{P}}_{k-1:k}$ is the set of matched features inliers.

The filtered matched feature set ${\hat{P}}_{k-1:k}$ can then be passed to any VO algorithm to estimate the incremental motion of the camera and to compute the odometry.

## 4. Experimental Work

To show the generic aspect of the proposed pipeline, simple stereo, RGB-D, and monocular VO algorithms are implemented for validation. The motion estimation techniques used are the same as those described in [7].

The algorithms are implemented in Python using OpenCV library, SURF for feature tracking, and the extracted features are matched between consecutive frames by Brute-Force matching. All experiments and tests were conducted on a computer with an Intel i7-8850H 6-core processor running at 2.60 GHz using 16 GB of RAM, running Ubuntu 16.04. Furthermore, the algorithms were implemented with a Robot Operating System (ROS) wrapper node to be compatible with the ROS framework [57].

The VO algorithms were then used to estimate the motion of the camera using sequences from KITTI [34], TUM [35], as well as experimental sequences generated by Summit-XL Steel manufactured by Robotnik Inc. [58]. The performance of the pipeline is evaluated through several comparisons, which demonstrate the effect of the added stages to the VO pipeline.

### 4.1. Motion Estimation

#### 4.1.1. Stereo/RGB-D Visual Odometry

The stereo and RGB-D VO algorithms used in this paper rely on solving the same 3D-to-2D correspondence problem. First, the features in the image $I_{k-1}$ along with the disparity map or the depth image are used to produce the 3D features $F_{k}$ in $I_{k-1}$. The motion of the camera is then estimated by solving the Perspective-n-Point (PnP) problem. The PnP problem is solved in a RANSAC scheme [8]. This is after utilizing the AOR to achieve better and more efficient motion estimation.

(8) $$T_{k-1:k}=\underset{T_{k-1:k}}{arg min}\sum_{i=0}^{N_{f}}{∥f_{k}^{i}-{\hat{F}}_{k-1}^{i}∥}_{2}^{2}$$

$T_{k-1:k}\in SE\left(3\right)$ is the transformation matrix describing the incremental motion between time-steps k−1 and *k*, $f_{k}^{i}$ is *i*-th 2D feature in the current image, ${\hat{F}}_{k-1}^{i}$ is the same feature in 3D, reprojected from image $I_{k-1}$ onto the current image $I_{k}$ through $T_{k-1:k}$, and $N_{f}$ is the total number of features in the image.

#### 4.1.2. Monocular Visual Odometry

To estimate the motion from a single camera, the Epipolar constraint between frames is used as

(9) $$\left[\begin{array}{cc} x_{k-1,i} & y_{k-1,i} \end{array}\right]E\left[\begin{array}{c} x_{k,i}\\ y_{k,i} \end{array}\right]=0,\;\;\forall \;0\leq i\leq N_{f}$$

where $E\in R^{3\times 3}$ is the essential matrix for the calibrated camera [16].

The essential matrix E is estimated using the five-point algorithm proposed in [59]. After obtaining the essential matrix, it is decomposed into the translation and rotation of the camera as described in [16]. Furthermore, the motion estimation algorithm is executed with the Least Median Of Squares (LMEDS) [60] scheme in order to achieve better motion estimation.

Using a monocular camera, the ego-motion of the camera can be estimated up to a scale. To compensate for this scale, a velocity measurement of the vehicle needs to be available through the use of an external sensor such as wheel encoders, an IMU, a GPS, or through the CAN data from the vehicle’s tachometer.

The implementation of the VO algorithms from scratch was intended for the ease of integration of the proposed pipeline. However, the pipeline, in general, can be integrated to any VO implementation.

### 4.2. Datasets

#### 4.2.1. KITTI Vision Benchmark Dataset

KITTI Vision Benchmark Suite was selected as a publicly available dataset [34]. The dataset provides ground-truth ego-motion for 11 training sequences and 11 test sequences. The ground-truth is provided as a list of 6D poses for the training sequences, whereas for the test sequences, evaluation results are obtained by submitting them to the KITTI website. The dataset is sampled at 10 Hz at an average speed of 90 km/h, which creates a challenge in using the dataset for training and testing. Sequence 3 from the training subset is no longer available, as it was removed by KITTI for its similarities with the test sequences.

The dataset comprises the following information: raw synced and rectified color images from the left and right cameras and raw 3D GPS/IMU unfiltered data, along with the timestamps for all recordings. In order to convert the raw data to ROS bagfiles, the kitti2bag package was used [61]. The dataset also provides a tool for evaluating the performance of the VO and visual SLAM algorithms. This tool was used in the paper to evaluate the proposed pipeline in the case of the KITTI dataset.

#### 4.2.2. TUM RGB-D Dataset

The TUM RGB-D dataset is a large dataset containing sequences captured by an RGB-D camera along with its ground-truth to establish a benchmark for evaluation of VO and visual SLAM algorithms [35]. The dataset contains the color and depth images taken by a Microsoft Kinect camera, while the ground truth was recorded using a high-accuracy motion capture system with eight high-speed tracking cameras (100 Hz). The data were recorded using a 30 Hz rate with a camera resolution of 640×460. The dataset also provides an online tool through which the results are submitted for evaluating the performance of VO and visual SLAM systems. In this paper, the TUM sequences are evaluated using the Relative Pose Error (RPE), which is recommended by the dataset for VO algorithms [62].

RPE is basically the error in relative motion between the pairs of the VO output. The evaluation tool by the TUM dataset computes the error between all pairs of the output and generates the evaluation metrics such as the Root Mean Square Error (RMSE), mean, max, etc. In this paper, the RMSE error for the translation and orientation is used for evaluation.

#### 4.2.3. Images from Omnidirectional Robot

**Figure 5:**
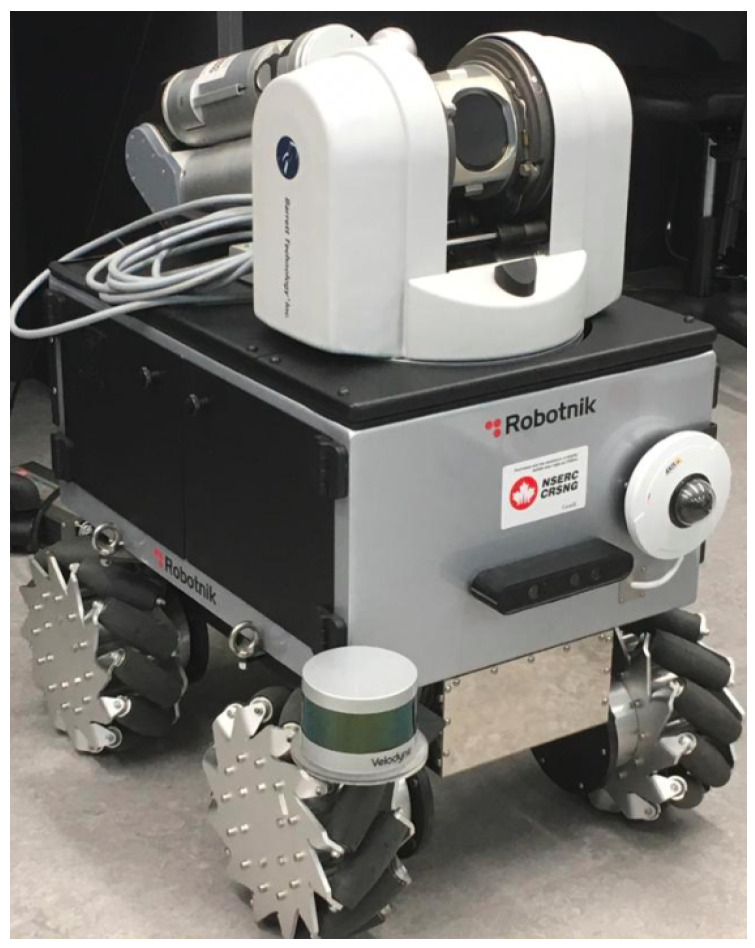
The omni-directional mobile robot used to validate the monocular VO algorithm with the proposed pipeline.

Summit XLS is a ground mobile robot with mecanum wheels, shown in Figure 5. The robot is equipped with an Astra RGB-D Camera (https://shop.orbbec3d.com/Astra, accessed on 27 October 2022), as well as wheel encoders. Several experiments were made using the robot to validate the proposed pipeline, while using the VICON motion capture system (https://www.vicon.com/, accessed on 27 October 2022) as a reference. The VICON system used consists of 12 cameras and the VICON bridge package was used to couple VICON with ROS [63]. Since the RGB-D VO case is tested and validated using the TUM dataset, the Summit-XL Steel sequences are used to validate the monocular VO while relying on the wheel encoders to obtain the speed for motion scaling. The evaluation is again conducted using the RMSE error for translation and orientation.

Three different sequences were executed using the robot in remote-control mode. In the first two sequences, the robot moved in semi-rectangular paths while, in the third sequence, the robot moved in a circular path. The total length of each of the paths was 12.5 m in the case of the rectangular paths and 6.5 m in the case of the circular path.

## 5. Results and Discussion

### 5.1. KITTI Vision Benchmark Dataset/Stereo VO

#### 5.1.1. Pose Accuracy Comparison

In order to show the efficacy of the proposed algorithm, the pose estimation results from a stereo VO are reported with and without the proposed pipeline. Table 1 shows the accuracy comparison using the 10 sequences available from the KITTI dataset. The results shown are the translation and rotation RMSE values generated by the dataset evaluation tool. As can be seen in the table, the pipeline enhanced the pose estimation accuracy in almost all the sequences.

**Figure 6:**
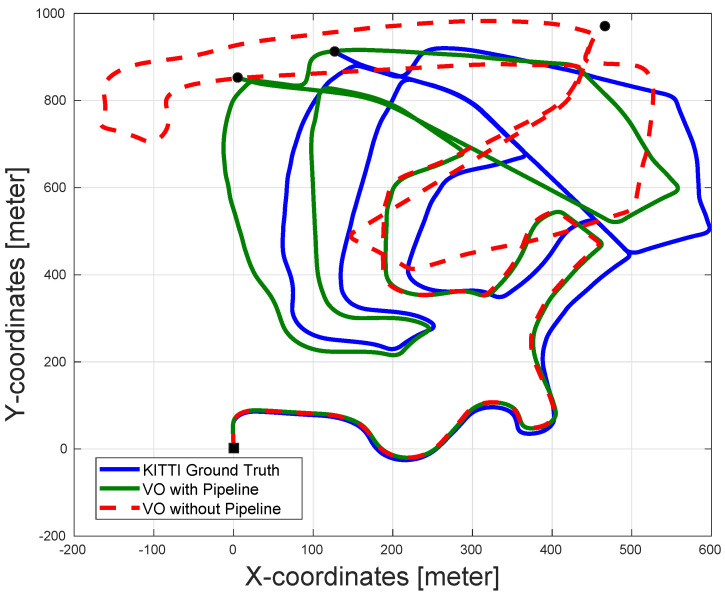
The pose estimation results of sequence 2 in the KITTI dataset with and without pipeline along with the dataset ground-truth. The proposed pipeline significantly enhances the output accuracy of the VO in this sequence. The VO output without the pipeline suffers from major drift errors and can even be considered to diverge. The black square represents the start of the paths and the black circles represent the ends of the paths.

In sequence 2 (shown in Figure 6), note that the effect of the pipeline is very obvious since the presence of the pipeline significantly enhanced the pose estimation accuracy compared to the VO pose estimation without the pipeline. Notice that the reason for the divergence of the VO in the first case is due to the absence of enough features in the images, which made the VO unable to estimate the incremental motion for long durations in the sequence. On the other hand, using the CLAHE filter increases the number of stable features in the images, while using the AOR algorithm along with the RANSAC (which is present in both cases) ensures more accurate incremental motion estimation for all received images. This leads to a much better VO output, as shown in Figure 6.

**Figure 7:**
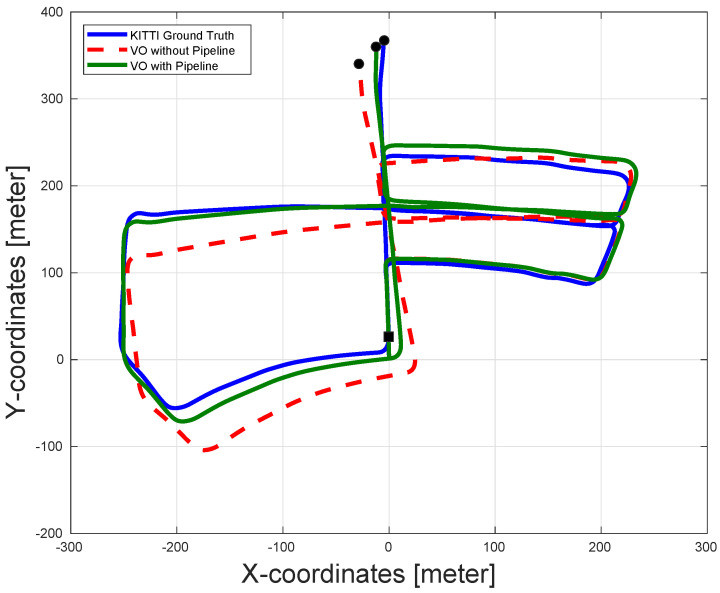
The pose estimation results of sequence 5 in the KITTI dataset with and without the pipeline, along with the dataset ground-truth. The performance of the VO with the pipeline enhances the performance of the VO in both translation and orientation estimation. This is not reflected in the average RMSE of the sequence due to the drift in the orientation of the VO without the pipeline, which causes some estimated poses to look close to the ground truth, although the overall estimated trajectory is much worse. The black square represents the start of the paths and the black circles represent the ends of the paths.

In sequence 5, although the average translation RMSE of the VO without the pipeline is lower than that with the pipeline, the actual performance of the pose estimation for the VO with the pipeline is much better (as shown in Figure 7). The real performance of the VO odometry is not reflected in Table 1 because the drift in the orientation of the VO without the pipeline causes some estimated poses to look closer to the ground truth compared to the VO output with the pipeline. However, the overall path estimated by the VO with the pipeline is superior to that of the VO without the pipeline.

**Figure 8:**
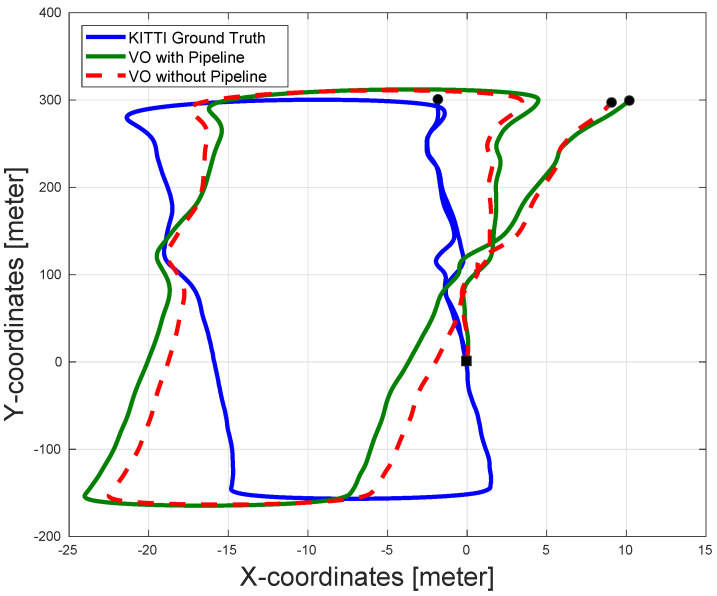
The pose estimation results of sequence 6 in the KITTI dataset with and without the pipeline along with the dataset ground-truth. The VO without the pipeline shows more accurate pose estimation compared to VO with the pipeline. However, the performance of both algorithms are very similar. The black square represents the start of the paths and the black circles represent the ends of the paths.

Finally, in the case of sequence 6, the performance of the VO without the pipeline outperformed that of the VO with the pipeline, as seen in Figure 8. This may be attributed to the fact that the amount of features available after applying the AOR is not enough for accurate motion estimation. This is further discussed in Section 5.1.2.

#### 5.1.2. Effect of AOR

One of the contributions of the paper is the new outlier rejection algorithm named AOR. In this subsection, the effect of AOR alone on the performance of the VO is studied. To this end, the translation and orientation RMSE results for the VO with AOR are also reported in Table 1.

As shown in Table 2, the AOR significantly contributed to the enhancement of some of the sequences. For example, the table shows that the use of AOR was responsible for the enhancement of sequence 2, which diverged without the use of it, as shown in Figure 6. Furthermore, the use of AOR also resulted in better results for sequences 0, 4, and 7.

The use of AOR resulted in worse results in the case of the other results. The reason for this effect is the absence of enough features for motion estimation after applying the AOR algorithm, which results in worse motion estimation due to the limited amount of information available. This problem can be addressed by increasing the threshold of the AOR η to increase the number of inliers.

As can be seen in the results, the AOR algorithm is an aggressive outlier rejection method. In other words, the AOR might result in the removal of matched features with slight deviations. This means that the use of AOR on an image requires the presence of a sufficient amount of stable features for motion estimation. Otherwise, the AOR will lead to the deterioration of the motion estimation due to decreasing the amount of matched features. The threshold weight can be altered to obtain a balanced amount of inliers to minimal outliers. However, this will improve the output of some sequences, and other situations might deteriorate. This is why the integration of AOR with CLAHE and SSC is a very good combination because each of the three stages plays a role in enhancing the motion estimation while complementing the negative effects of the other ones.

Although the use of AOR can lead to the removal of many matched features, the presence of CLAHE increases the number of stable features in the images. Moreover, despite the increase in features due to CLAHE, this might lead to a concentration of features in a certain region of the image. However, the use of SSC prevents such poor distribution of the features. Although the use of CLAHE, while adding more stable features, can also add more outliers in the features, the AOR acts to remove these outliers. In conclusion, the presence of the three stages of the pipeline acts as a desirable combination to overcome the drawbacks of each of the stages while utilizing their advantages.

It is worth mentioning that the AOR resulted in worse results for some sequences; however, the overall performance of the VO with the pipeline (2.71%) was better than that of the VO without the pipeline (3.29%). Furthermore, the use of the complete pipeline still resulted in better accuracy for almost all sequences compared to the VO without the pipeline, as shown in Table 1.

The VO with pipeline results in better performance because the use of CLAHE increased the overall amount of features while SSC uniformly distributed those features helping to achieve better results along with the AOR outlier removal.

#### 5.1.3. Computational Cost

It is expected that adding processing stages to the VO algorithm will lead to an increase in computational time. This can indeed be seen in Table 3, where the average computation time for the VO is reported after adding each of the proposed stages of the pipeline. However, the significant benefits in the accuracy of the pose estimation outweigh the increased computational cost.

In Table 3, the computation time of the VO algorithm with the AOR algorithm only is reported. Notice that the computation time of the algorithm after adding the AOR to the VO is less than that of the VO without AOR. As discussed in Section 3, this is due to the fact that the AOR removes the false-matched features. Accordingly, this simplifies the mission of the RANSAC, which results in a faster motion estimation convergence and a faster performance, as shown in Table 3. This also explains why the full pipeline computational time is less than the case of adding CLAHE only or CLAHE and SSC.

The mentioned average computational time shows that the algorithm is capable of working in real-time while receiving up to 6 fps. The performance of the algorithm, as well as the accuracy of the pose estimation, can be further enhanced through the use of a Graphical Processing Unit (GPU) and multithreading processing.

### 5.2. TUM RGB-D Dataset/RGB-D VO

#### 5.2.1. Pose Accuracy Comparison

Table 4 shows the translation and orientation RMSE for nine sequences from the TUM RGB-D dataset. As shown in the table, the RGB-D VO with the proposed pipeline shows better pose estimation accuracy for all sequences. This enhancement varies from one sequence to another based on the lighting and the number of features available in each sequence.

**Figure 9:**
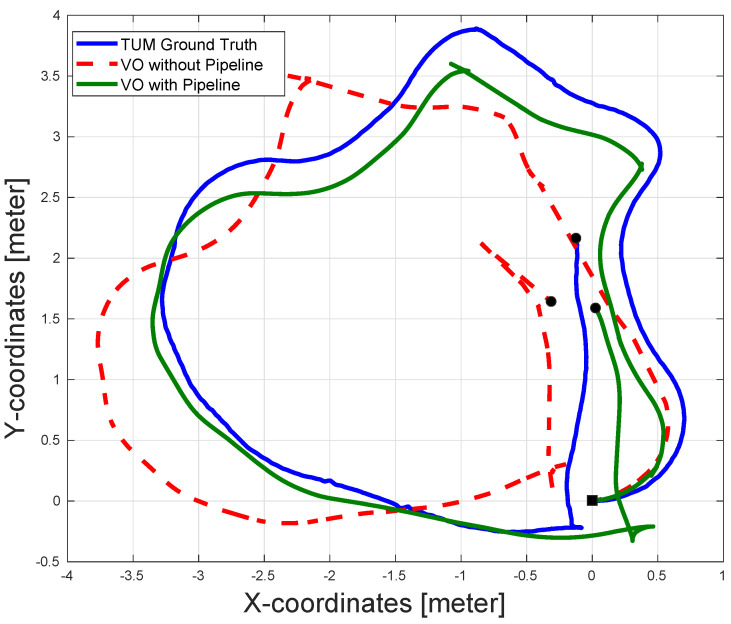
The pose estimation results for the sequence fr1/pioneer_360 in the TUM dataset with and without the pipeline along with the dataset ground-truth. The pose estimation accuracy of the VO with the pipeline is superior, while the VO without the pipeline suffers from both poor translation and orientation estimation. The black square represents the start of the paths and the black circles represent the ends of the paths.

Figure 9 shows an example of the pose estimation output from the VO algorithm with and without the pipeline. It can be seen that the VO without the proposed pipeline suffers from large motion estimation errors at the beginning of the path, which in turn results in large drift errors over the rest of the path. As for the VO with the pipeline, although there is still an error in the estimated path, the error is significantly smaller than that of the VO without the pipeline, especially at the beginning of the path, causing a much better estimation for the rest of the path. The better performance of the VO with the pipeline algorithm is attributed to the increased amount of detected features at the beginning of the path compared to that of the VO without the pipeline.

**Figure 10:**
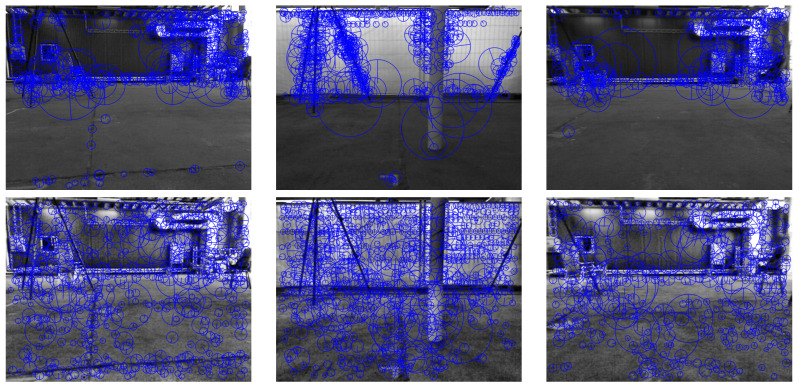
Three examples from TUM sequences showing the effect of CLAHE and SSC in indoor scenarios. The images in the top row show the images with the detected features using SURF, detected without the use of CLAHE and SSC algorithms. The images in the bottom row show the features detected by SURF after adding the CLAHE and SSC stages.

Since the TUM sequences are recorded indoors, adding the CLAHE stage results in a significant increase in the detected features (some examples are shown in Figure 10). Note that for these sequences, an RGB-D VO algorithm is used, which means that only the features with an observable depth by the depth sensor can be used for motion estimation. In other words, the far features in the images cannot be used. Through the SSC algorithm, the features detected in the images are well distributed, and thus, the number of close features with observable depth increases (see Figure 10).

The results shown in Figure 9 and Table 4 confirm the efficacy of the proposed pipeline for VO algorithms, even for indoor scenarios.

#### 5.2.2. Computational Cost

Table 5 shows the average computational time for the VO with the different added stages. Notice that, in this case, the results are slightly different from those of the computation cost analysis shown for KITTI sequences. As expected, the computational cost increases for the different stages of the proposed pipeline. However, in KITTI sequences, adding the AOR algorithm to the pipeline resulted in a faster performance compared to the VO without any stages. This is not the case for TUM sequences, where the computational cost still increases with the AOR algorithm. It is postulated that the amount of features detected in the case of TUM is larger or the number of outliers in the matched features set is larger, which is mainly based on qualitative and quantitative analysis. Nevertheless, adding the AOR to the pipeline results in lowering the computation cost of the VO algorithm. As can be seen in Table 5, the average computational cost of the VO algorithm when adding CLAHE and SSC is larger than that of the overall pipeline computational cost.

### 5.3. Summit XL Steel Sequences/Monocular VO

#### 5.3.1. Pose Accuracy Comparison

The translation and orientation RMSE are reported in Table 6 for three different scenarios. The results show the accuracy enhancement in the case of the VO with the pipeline. For the Summit XL Steel robot sequences, a monocular VO algorithm was used for validating the proposed processing pipeline. Since the scale is unobservable, the robot’s wheel encoders are used to calculate its speed and the scale of the odometry. This means that a component of the error is due to the error in the velocity measurement taken by the encoders. However, since the same data are used for both cases, this effect is the same for both cases and will not make any bias in the comparison.

**Figure 11:**
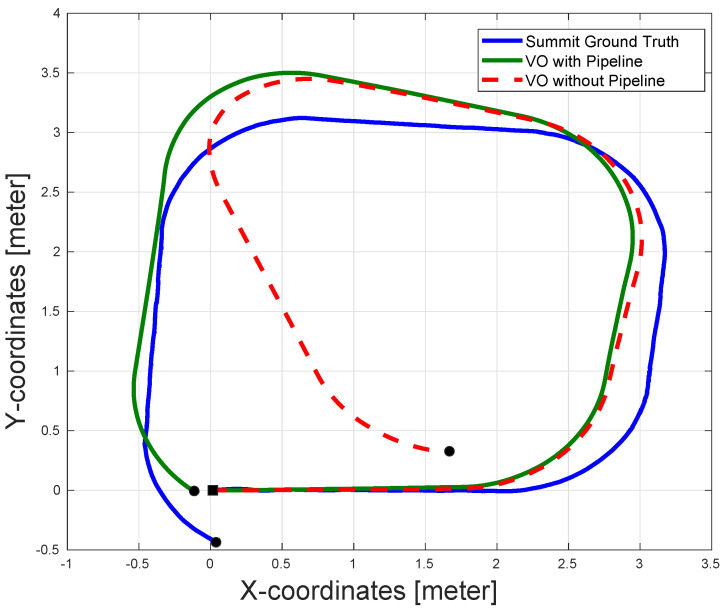
The pose estimation results for a rectangular sequence executed by Summit XLS steel. The figure shows the output of the VO with and without the proposed pipeline. The VO with the pipeline shows better pose accuracy especially at the end of the sequence while the VO without the pipeline suffers from significant amount of drift error. The black square represents the start of the paths and the black circles represent the ends of the paths.

Figure 11 shows the estimation results for VO with and without the pipeline. As can be seen in the figure, the accuracy is superior in the case of the VO with the proposed pipeline. This is especially true at the end of the sequence, at which the VO without the pipeline suffered from a large drift error. The presence of the AOR resulted in removing many matched outliers, which would have caused bad motion estimation and a significant amount of drift errors in the case of VO without the proposed pipeline.

#### 5.3.2. Computational Cost

Table 7 shows the computational time analysis of several combinations of VO algorithms, including the proposed VO algorithm. As was illustrated before, the best computational performance was for the VO with the AOR algorithm. This is a direct result of the better outliers removal of the AOR, which results in a better and faster convergence of the motion estimation algorithm. The table also shows the translation and orientation RMSE for each of the different VO combinations. The results confirm that the proposed VO results in the best performance.

### 5.4. Discussion

For all the scenarios, and for all VO types used in this paper, the proposed pipeline showed better performance compared to the VO without the pipeline. Specifically, adding the three additional stages to the actual VO algorithm enhanced the accuracy by an average of 37% for the considered datasets. These additional three stages can be added to any feature-based VO algorithm to enhance its accuracy and robustness.

In Table 3, Table 5 and Table 7, the results for different combinations of the three proposed stages were reported. In the three cases, the VO with the full proposed pipeline showed better pose estimation accuracy. This shows that the three stages proposed in this paper are integral. Each one of the three serves its own purpose and contributes to the overall enhancement. However, as expected, this came with an increase in the computational cost of the algorithm. Notice that the increase in the computational cost is still not significant (an average of 37 ms) and does not result in a large reduction in the number of frames per second. In most applications, this increase in the computational cost can be accepted to improve the pose estimation accuracy in return (as shown in Table 8).

## 6. Conclusions and Future Works

In this paper, an image processing pipeline was introduced to enhance the accuracy and robustness of VO algorithms. The proposed pipeline consists of three stages, CLAHE, SSC, and AOR. Each stage addresses a separate issue associated with pose estimation error.

The proposed pipeline is intended to be generic and modular, which can be embedded in any feature-based algorithm in order to enhance its performance. In order to validate the proposed pipeline, sequences from KITTI and TUM datasets, as well as experimental sequences generated by a commercial omnidirectional mobile robot, were used. For each dataset, one type of VO was used for validation, namely stereo, RGB-D and monocular. The quantitative and qualitative results show that the proposed pipeline has a significant enhancement in the VO accuracy and robustness, with a minor increase in the computational time.

As mentioned earlier, a VO algorithm relies on integration and, consequently, can suffer from large amounts of errors or the overall divergence of the pose estimation through operation. This can occur due to several causes, such as poor lighting conditions, false-matched features or motion bias. Throughout this paper, the three aforementioned causes of error were addressed by designing a generic pipeline that can be integrated into any visual odometry algorithm to enhance its accuracy.

As a future work, the proposed pipeline is planned to be integrated into visual SLAM algorithms, and the effect of the pipeline will be studied. Furthermore, comparisons with deep learning approaches will also be conducted to see which approach works best with which conditions. Several additional filtration steps will also be investigated to further enhance the performance of VO algorithms. Meanwhile, the computational cost of the algorithm is expected to be reduced through the use of GPUs and parallel computing techniques.

## Figures and Tables

**Table 1:** KITTI dataset accuracy comparison.

Sequence Number	VO without Pipeline		VO with AOR		VO with Pipeline	
	Tr (%)	Rot (\deg/m)	Tr (%)	Rot (deg/m)	Tr (%)	Rot (deg/m)
0	1.85	0.011	1.80	0.011	**1.68**	**0.011**
1	5.14	0.013	5.33	0.013	**4.72**	**0.012**
2	6.99	0.036	**1.82**	**0.016**	1.89	0.017
4	3.91	0.007	2.75	**0.006**	**0.33**	0.007
5	**2.38**	0.011	2.75	0.012	2.43	**0.009**
6	**2.62**	0.010	2.74	0.010	2.88	**0.010**
7	2.11	0.016	**1.95**	**0.014**	2.07	0.015
8	2.57	0.012	2.65	**0.011**	**1.92**	0.012
9	2.70	0.019	2.86	0.019	**1.78**	**0.019**
10	2.65	0.020	2.82	0.02	**2.02**	**0.020**
Overall	3.29	0.015	2.71	0.013	**2.18**	**0.013**

**Table 2:** AOR effect on pose estimation.

Seq. No.	VO without AOR		VO with AOR	
	Tr (%)	Rot (deg/m)	Tr (%)	Rot (deg/m)
0	1.85	0.011	**1.80**	0.011
1	**5.14**	0.013	5.33	**0.013**
2	6.99	0.036	**1.82**	**0.0158**
4	3.91	0.007	**2.75**	**0.0056**
5	**2.38**	**0.011**	2.75	0.012
6	**2.62**	0.010	2.74	0.010
7	2.11	0.016	**1.95**	**0.014**
8	**2.57**	0.012	2.65	**0.011**
9	**2.70**	0.019	2.86	0.019
10	**2.65**	0.020	2.82	0.020
Overall	3.29	0.015	**2.71**	**0.013**

**Table 3:** Computation time comparison for KITTI sequences.

VO Type	Comp. Time (mean ± std [ms])	Tr RMSE (%)	Rot RMSE (deg/m)
Vanilla	116 ± 31	3.29	0.0154
CLAHE	176 ± 36	2.88	0.0136
CLAHE and SSC	181 ± 34	2.86	0.0136
AOR Only	**106 ± 24**	2.71	0.0134
Full Pipeline	160 ± 35	**2.18**	**0.0133**

**Table 4:** TUM accuracy comparison.

Seq. Name	VO without Pipeline		VO with Pipeline	
	Tr (m)	Rot (deg)	Tr (m)	Rot (deg)
fr1/xyz	0.24	8.80	**0.04**	**2.08**
fr1/desk	0.43	19.5	**0.07**	**3.55**
fr1/desk2	0.46	23.8	**0.09**	**6.74**
fr1/room	0.32	23.5	**0.12**	**5.73**
fr2/pioneer_360	0.18	6.50	**0.10**	**3.58**
fr2/pioneer_slam	0.10	3.60	**0.09**	**2.38**
fr2/pioneer_slam2	0.07	2.82	**0.07**	**2.00**
fr2/pioneer_slam3	0.07	2.03	**0.05**	**1.44**
fr2/desk	0.19	5.20	**0.02**	**0.64**
Overall	0.23	10.6	**0.07**	**3.12**

**Table 5:** Computation time comparison for TUM sequences.

VO Type	Comp. Time (mean ± std (ms))	Tr RMSE (m)	Rot RMSE (deg)
Vanilla	**118 ± 26**	0.229	10.6
CLAHE	179 ± 48	0.213	10.7
CLAHE and SSC	185 ± 53	0.210	9.98
AOR Only	169 ± 36	0.135	10.4
Full Pipeline	176 ± 40	**0.073**	**3.13**

**Table 6:** Summit accuracy comparison.

Seq. Name	VO without Pipeline		VO with Pipeline	
	Tr (m)	Rot (deg)	Tr (m)	Rot (deg)
Rectangle 1	0.49	1.67	**0.34**	**1.51**
Rectangle 2	0.25	1.69	**0.25**	**1.52**
Circle	0.50	2.09	**0.48**	**2.07**
Overall	0.31	1.75	**0.27**	**1.67**

**Table 7:** Computation time comparison for summit XLS steel sequences.

VO Type	Comp. Time (mean ± std (ms))	Tr RMSE (m)	Rot RMSE (deg)
Vanilla	148 ± 28	0.315	1.68
CLAHE	165 ± 47	0.350	1.68
CLAHE and SSC	168 ± 42	0.302	1.74
AOR Only	**132 ± 19**	0.287	1.68
Full Pipeline	158 ± 34	**0.275**	**1.67**

**Table 8:** The pipeline effect on VO.

Dataset	Tr (%)	Rot (%)	Comp. Time (ms)
KITTI	−33%	−13%	+44
TUM	−68%	−70%	+58
Summit	−12%	−0.5%	+10

## Data Availability

Publicly available datasets were analyzed in this study. This data can be found here: [https://vision.in.tum.de/data/datasets/rgbd-dataset, www.cvlibs.net/datasets/kitti, accessed on 27 October 2022].

## Abbreviations

The following abbreviations are used in this manuscript:

MDPI	Multidisciplinary Digital Publishing Institute
DOAJ	Directory of Open Access Journals
TLA	Three Letter Acronym
LD	Linear Dichroism
